# A Comparative Study of the Effects of Cholesterol and Lanosterol on Hydrated Phosphatidylethanolamine Assemblies: Focusing on Physical Parameters Related to Membrane Fusion

**DOI:** 10.3390/membranes15120352

**Published:** 2025-11-24

**Authors:** Ayumi Okayama, Michael Postrado, Hiroshi Takahashi

**Affiliations:** Division of Pure and Applied Science, Faculty of Science and Technology, Gunma University, 4-2 Aramaki, Maebashi 371-8510, Gunma, Japan

**Keywords:** cholesterol, lanosterol, phosphatidylethanolamine, membrane fusion, molecular volume, inverted hexagonal phase, interbilayer interaction, X-ray diffraction, electron density profile

## Abstract

Cholesterol (Chol) plays a crucial role in regulating membrane properties and biological processes such as membrane fusion, yet the molecular mechanisms underlying its function remain incompletely understood. In order to elucidate how sterol structure influences phospholipid organization relevant to membrane fusion, we compared the effects of Chol and its biosynthetic precursor lanosterol (Lan) on hydrated phosphatidylethanolamine (PE) assemblies using X-ray diffraction, the neutral flotation method, and osmotic stress measurements. Volumetric analyses revealed that Lan has a larger occupied molecular volume than Chol in the bilayers. These values were largely independent of differences between phospholipids (phosphatidylcholine and PE), indicating that sterols are deeply embedded within the bilayer. In palmitoyl-oleoyl-PE lamellar membranes, both sterols increased bilayer thickness. They both enhanced short-range repulsive hydration forces, but Chol suppressed fluctuation-induced repulsion more effectively, reflecting its greater stiffening effect. In bacterial PE systems forming the inverted hexagonal (H_II_) phase, increasing sterol concentration decreased the lattice constant, with a more substantial effect for Lan, which also induced greater curvature of the water columns. These results suggest that while Chol enhances mechanical rigidity and membrane cohesion, Lan promotes molecular flexibility and curvature, properties associated with fusion intermediates.

## 1. Introduction

Cholesterol (Chol) is the most abundant sterol found in the cell membranes of mammals [1,2,3,4]. As well as serving as a precursor for hormone synthesis, it is involved in various physiological functions [1,2,3,4]. It provides an optimal environment for membrane proteins to function by regulating membrane fluidity, rigidity, and thickness [5,6]. It also contributes to the barrier properties of membranes, influencing the permeability or entry of small molecules according to the concentration of Chol present [7,8,9,10,11,12]. Chol plays an indispensable role in the formation of lipid rafts, which are essential membrane domains for signal transduction [13,14,15]. The relationship between cancer and lipid rafts has also been actively researched in recent years [16]. In addition, as described below, recent years have seen growing attention focused on the role of Chol in membrane fusion processes.

Membrane fusion is a fundamental biological process that underlies diverse phenomena such as neurotransmitter release [17] and viral infection [18]. For example, severe acute respiratory syndrome coronavirus 2 (SARS-CoV-2) undergoes membrane fusion during host cell entry and upon release after replication [19,20]. Although membrane proteins are directly involved in fusion [21], it is the lipid bilayer that provides the structural basis for this process [22]. Numerous studies have suggested that Chol plays a critical role in this infection process [20]. Clinical observations initially reported that patients taking statins, cholesterol-lowering drugs, exhibited reduced severity and mortality [23]. Subsequent cellular studies demonstrated that SARS-CoV-2 infection is Chol-dependent [24]. Chol involvement has also been reported in infections by other viruses, including Ebola [25] and influenza [26]. However, the molecular mechanisms by which Chol influences membrane fusion remain largely unresolved [19].

Chol is synthesized in vivo from acetic acid through a series of chemical reactions, including oxidation steps [27]. Konrad Bloch, who elucidated this biosynthetic pathway and was awarded the Nobel Prize, proposed that Chol biosynthesis represents an evolutionary refinement of molecular function [28,29,30]. Indeed, Lan—the immediate precursor of Chol—cannot fully substitute for Chol in terms of its biological functions or its effects on the physical properties of lipid bilayers. The growth rate of *Mycoplasma capricolum* is markedly reduced when the cells are supplied with Lan as the sole sterol, instead of Chol [30,31]. Biophysical studies using artificial model membrane systems have shown that, compared with Chol, Lan is less effective at densely packing lipid molecules within the bilayer, at increasing membrane rigidity, and at inducing phase separation to form sterol-rich membrane domains [32,33,34,35,36,37,38,39,40,41,42,43,44,45]. These findings indicate that even minor structural differences can significantly influence the physical properties of membranes. Lan differs from Chol by the presence of three additional methyl groups on its steroid ring.

In our previous work [45], we prepared artificial membranes composed of 1-plmitoyl-2-oleoyl-*sn*-glycero-3-phosphocholine (POPC), a major phospholipid in the outer leaflet of the plasma membrane [4], mixed with either Chol or Lan, and compared their physical properties. We also found that membranes containing Chol are more tightly packed and thicker, suggesting that Chol provides superior mechanical strength and barrier function compared to Lan.

While POPC mimics the outer leaflet of the plasma membrane, membrane fusion also occurs in the inner leaflet, which contains high levels of phosphatidylethanolamine (PE) [4]. When enveloped viruses are released from infected cells, they undergo membrane fusion with the inner leaflet of the plasma membrane. Therefore, we consider it meaningful to research the mechanism of membrane fusion using artificial membrane systems containing PE.

The membrane fusion process during SARS-CoV-2 infection proceeds as follows [18]: (1) the spike protein of the coronavirus binds to a cellular receptor; (2) a host enzyme hydrolyzes a portion of the spike protein; (3) this exposes a hydrophobic region; (4) the hydrophobic region then penetrates the host cell membrane; (5) the protein subsequently undergoes a conformational change that brings the viral envelope and the host cell membrane into close proximity; and (6) membrane fusion occurs. The detailed mechanisms underlying steps (5) and (6) remain incompletely understood. Step (6) is believed to involve the transient formation of an inverted micelle-like structure. Because of its small hydrophilic headgroup, PE readily undergoes a phase transition from the lamellar phase to the inverted hexagonal (H_II_) phase, which resembles an inverted micellar structure. Numerous studies on artificial membrane systems have shown that factors promoting the formation of the H_II_ phase also facilitate membrane fusion [46,47,48,49,50,51,52,53].

Two key questions arise: (a) How does the addition of sterols influence H_II_ phase formation in PE membranes? For membranes to approach each other during step (5), water molecules must first be removed. The strong interactions between water molecules and the polar headgroups of lipid bilayers (known as hydration repulsive forces [54,55,56]) make such close approach difficult. (b) How does the addition of sterols affect interfacial interactions in PE membranes? These questions remain insufficiently explored. Furthermore, what would happen if Lan were added instead of Chol?

The aim of this study is to elucidate the effects of Chol and Lan on the interbilayer interactions during the initial approach of two membranes, as well as on the structure of the H_II_ phase in PE-based systems. In this study, we used two PE species, synthetic 1-Palmitoyl-2-oleoyl-*sn*-glycero-3-phosphoethanolamine (POPE) and natural PE extracted from the bacterium *Escherichia coli* (BPE).

In addition, using the neutral flotation method, we also measured the mass density of each sterol in the lamellar phase and calculated its molecular volume. The average molecular volume of Lan was found to be significantly larger than that of Chol. Interestingly, these values were consistent across both POPC and POPE systems, suggesting that sterols are deeply embedded within the membrane’s hydrophobic region and that their occupied volume is mainly independent of the phospholipid headgroup type.

The addition of sterols enhanced the short-range repulsive force between membranes at close proximity. However, Chol and Lan exhibited distinct behaviors: the repulsive force arising from membrane fluctuations [57,58,59] was more strongly suppressed by Chol, as it stiffens the membrane to a greater extent than Lan. X-ray diffraction experiments revealed that increasing sterol concentration reduced the lattice constant of the H_II_ phase, with the effect more pronounced for Lan than for Chol. Electron density reconstruction further showed that the curvature of the central water column in the H_II_ phase increased with rising sterol concentration, while the thickness of the hydrocarbon chain region remained nearly constant. Based on these findings, we discuss the roles of sterols in membrane fusion.

## 2. Materials and Methods

### 2.1. Chemicals

1-Palmitoyl-2-oleoyl-*sn*-glycero-3-phosphoethanolamine (POPE, purity ≥ 99%) and L-α-phosphatidylethanolamine from the bacterium *Escherichia coli* (BPE) were purchased from Avanti Polar Lipids, Inc. (Alabaster, AL, USA). According to the supplier, the average molecular weight of BPE is 719.302, with major acyl chain components of 16:0 and 18:1, and minor components of 14:0, 16:1, and 19:0. This average molecular weight was used for molar ratio calculations with sterols. Lanosterol (Lan, >99.5%) and cholesterol (Chol, 3β-cholest-5-en-3-ol, >99%) were obtained from Nagara Science Co., Ltd. (Gifu, Japan) and Sigma-Aldrich Co. LLC (St. Louis, MO, USA), respectively. Heavy water (D_2_O, 99.75%) for the neutral flotation method and polyvinylpyrrolidone (PVP, average molecular weight 40,000 g/mol) for the osmotic method were purchased from Fujifilm Wako Pure Chemical Co. (Osaka, Japan) and Sigma-Aldrich Co. LLC, respectively. All chemicals were used without further purification. Ultrapure water (light water, H_2_O) was prepared using a Milli-Q system (Millipore Corp., Bedford, MA, USA).

### 2.2. Preparation of Hydrated Samples

Sample preparation was performed almost identically to the method described in detail elsewhere [45]. The only difference was the hydration temperature. In brief, the sterol and phospholipid mixtures were prepared by mixing stock solutions dissolved in chloroform. Then, the chloroform was evaporated under a nitrogen stream, and the mixture was dried under vacuum. The dried sample obtained by this method was then hydrated by adding water. During hydration, the sample was heated to 40 °C and maintained at that temperature for approximately ten minutes.

The sample subjected to osmotic pressure from PVP was prepared as follows: Approximately 1 mL of pure water was added to about 4 mg of dried lipid mixture, which was then heated above 40 °C to hydrate and form multilamellar vesicles. The sample solution containing the multilamellar vesicles was centrifuged to precipitate them, after which the supernatant water was removed. Approximately 2 mL of PVP aqueous solution—an amount sufficiently large relative to the remaining water—was then added, and the mixture was heated to 40 °C and stirred again. The precipitated or floating sample was subsequently collected by centrifugation and placed in a glass capillary for X-ray diffraction measurement. When the PVP concentration is high, the mass density of the PVP aqueous solution exceeds that of the lipid sample. In this case, the lipid sample gathers at the top during centrifugation.

### 2.3. Volumetric Measurements

This measurement method has also been described in detail elsewhere [45], so we will only briefly mention it here. The mass density (ρ) of lipid systems was determined by the neutral flotation method using H_2_O/D_2_O mixtures [60,61] at 38 °C. For binary phospholipid/sterol vesicles, the apparent molecular weight was defined as $M_{ap}=X_{st}M_{st}+\left(1-X_{st}\right)M_{PL}$, and the apparent molecular volume as $V_{ap}\left(X_{st}\right)=M_{ap}/{\rho N}_{A}$, where $X_{st}$ is the sterol molar fraction, $M_{st}$ and $M_{PL}$ are the molecular weights of sterol and phospholipid, respectively, and $N_{A}$ is Avogadro’s number.

### 2.4. X-Ray Diffraction Measurements

X-ray diffraction experiments were conducted at the synchrotron radiation facility of the High Energy Accelerator Research Organization (KEK) (Tsukuba, Japan). Measurements of the POPE/sterol-based lamellar phase samples were conducted at the PF-BL10C station [62], while measurements of the BPE/sterol-based H_II_ phase samples were performed at the PF-BL6A station [63]. Both stations employ the same sample temperature control system. The hydrated sample was sealed in a glass capillary tube (Hilgenberg GmbH, Germany) with an outer diameter of 1 mm and a wall thickness of 10 μm. To suppress evaporation, the tube opening was flame-sealed. The capillary tube containing the sample was mounted onto a temperature-controlled stage (HCS302-LN190, Instec Inc., Boulder, Colorado, USA) using specially manufactured aluminum metal parts. X-ray diffraction patterns were recorded using the photon-counting pixel array detectors PILATUS1M (BL6A) or PILATUS2M (BL10C) (DECTRIS, Switzerland). The wavelengths (λ) of the X-ray beams used and detector-to-sample distances were 0.15 nm and ~900 mm for BL-6A and 0.10 nm and ~500 mm for BL-10C, respectively. The distance from the detector to the sample was precisely estimated by back-calculating from the diffraction peak positions of silver behenate, which was used as a standard sample [64]. The two-dimensional diffraction data were converted to one-dimensional data (Q vs. Intensity) using the Fit2D application [65] via circumferential integration. The Q value is defined as Q=4πsin2θ/λ, where 2θ is the scattering angle. The two-dimensional diffraction patterns were examined to ensure isotropy. Only the isotropic data were converted to one-dimensional data for analysis. Figure S1 in the Supplementary Materials shows two typical examples of two-dimensional diffraction pattern images.

### 2.5. Reconstruction of the Electronic Density Profiles

The relative one- and two-dimensional electron density profiles were calculated from the intensities of Bragg reflections observed in the lamellar and H_II_ phases, respectively. Here, let h and $\left(h,k\right)$ denote the Miller indices, and $F\left(h\right)$ and $F\left(h,k\right)$ represent the corresponding structure factors for the lamellar and H_II_ phases, respectively. Because the samples analyzed here were non-oriented specimens, the absolute values of the structure factors for each reflection were determined from the measured diffraction intensities after applying appropriate correction factors, including the Lorentz factor.

To compute the electron density distribution, phase information—specifically, the sign (+ or –) of each structure factor—is required. For the POPE/sterol system, the phase signs were determined using the so-called swelling method [66,67]. For the H_II_ phase, an electron density profile consistent with the expected H_II_ phase structure, as inferred from the chemical structure, was selected. In this task, we also referred to the electron density profiles of the H_II_ phase structure of PE already reported in the literature [68,69,70,71,72,73,74,75].

The one-dimensional relative electron density distribution in the lamellar phase was calculated as:

(1) $$\rho_{rel}\left(X\right)=\sum_{h}F\left(h\right)cos\left(\frac{2\pi hX}{d}\right),$$

where d is the lamellar repeat spacing and X is a one-dimensional coordinate position in real space.

In the H_II_ phase system, the two-dimensional relative electron density profile was calculated as:

(2) $$\rho_{rel}\left(x,y\right)=\sum_{h,k}F\left(h,k\right)cos\left[2\pi \left(hx+ky\right)\right],$$

where x and y are position coordinates within the unit cell. Using the relation $\left(X,Y\right)=\vec{r}=x\vec{a}+y\vec{b}$, where $\vec{a}$ and $\vec{b}$ are the unit cell basis vectors, the profiles were converted to real-space coordinates $\left(X,Y\right)$, and the resulting electron density maps are presented in real space.

## 3. Results

### 3.1. Molecular Volumes

**Figure 1:**
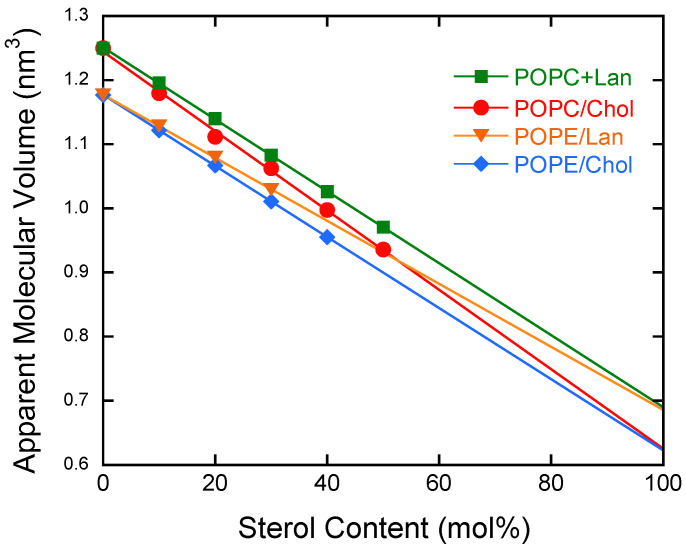
Plots of apparent molecular volume as a function of sterol content. POPE/Chol, (sky blue diamond shape), POPE/Lan, (orange inverted triangle), POPC/Chol (red circle), and POPC/Lan (green square). The data of PC systems are taken from [45].

Figure 1 shows the apparent molecular volumes of the POPE/sterol systems plotted against sterol concentration. For reference, previously reported data for the POPC/sterol systems at 25 °C [45] are also included.

The molecular volume of lipids was determined using the neutral flotation method with heavy water (D_2_O) and light water (H_2_O) [60,61]. Typically, the mass density of lipids lies between those of heavy and light water. This method involves observing the buoyancy and sedimentation behavior of lipid samples in two solutions of different concentrations. Identifying the concentration at which the sample neither floats nor sinks, but instead matches the solvent’s density, allows one to calculate the lipid sample’s mass density. Since the sample’s average molecular weight is known, the apparent average molecular volume can be estimated from the mass density determined in this manner. The experiments for the POPE/sterol system were conducted at 38 °C. At this temperature, pure POPE is in the fluid (L_α_) phase [68,69]

Within the concentration range measured in this study, the apparent molecular volume decreased linearly with increasing sterol content. Therefore, fitting this data with a linear function allows the occupied molecular volume of sterols in the lipid bilayer membrane to be determined by extrapolation to 100% sterol concentration.

Interestingly, the extrapolated values were almost identical for both the POPC and POPE systems. These values indicate the volume occupied by a single molecule of these sterols within the lipid bilayer. The occupied volumes of cholesterol (Chol) and lanosterol (Lan) in POPE bilayers at 38 °C were 0.623 nm^3^ and 0.686 nm^3^, respectively. As reported previously, the corresponding values in POPC bilayers at 25 °C were 0.617 nm^3^ and 0.691 nm^3^ [45]. Given the temperature difference and experimental uncertainties, the two sets of values are in good agreement.

Thus, the present experimental results indicate that the chemical difference in the polar heads does not significantly affect the apparent molecular volumes of the sterols (Chol and Lan) investigated here in lipid bilayer membranes.

### 3.2. POPE/Sterol System: Lamellar Structure, Thickness, and Molecular Area

**Figure 2:**
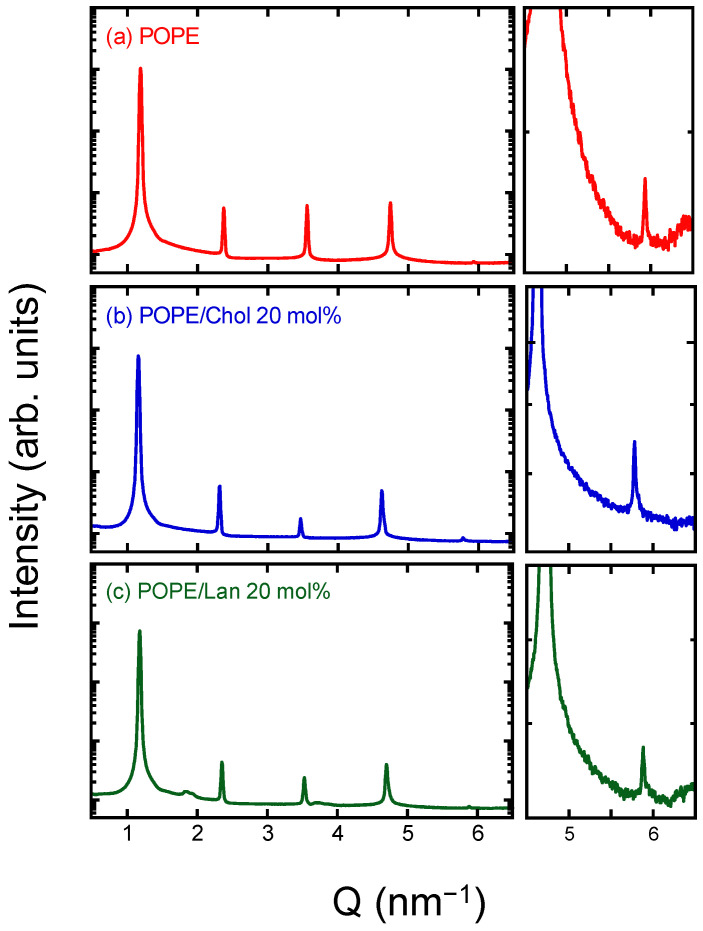
X-ray diffraction patterns of (**a**) pure POPE, (**b**) POPE/Chol (20 mol%), and (**c**) POPE/Lan (20 mol%). The patterns were recorded at 38 °C. For *Q* = 4.5–6.5 nm^−1^, enlarged patterns are also shown to facilitate recognition of small diffraction peaks.

Figure 2 shows the diffraction data obtained from X-ray diffraction experiments for pure POPE and POPE samples containing 20 mol% sterol. This diffraction experiment was also conducted at 38 °C, as in the experiment above. Because the samples consist of multilamellar vesicles, Bragg reflections (lamellar diffraction) corresponding to the lamellar periodicity were observed at regular intervals along the scattering vector *Q*. Note that the fifth-order reflection is very weak; therefore, an enlarged view of this region is provided.

The lamellar repeat distances were 5.23 nm for pure POPE, 5.40 nm for POPE/Chol (20 mol%), and 5.34 nm for POPE/Lan (20 mol%), respectively. The value obtained for pure POPE agrees well with those reported previously in the literature [68,69].

**Figure 3:**
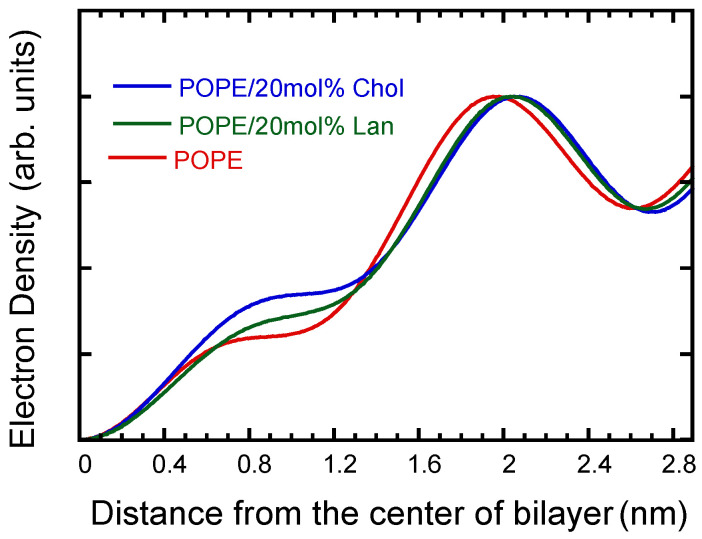
Relative electron density profiles reconstructed from the lamellar diffraction intensity data for pure POPE (red line), POPE/Chol **(**blue line), and POPE/Lan (green line).

The electron density profiles were reconstructed from the diffraction intensities of the observed peaks up to the fifth order, as shown in Figure 3. Phase determination required for the electron density calculation was carried out using the swelling method with the neutral, water-soluble polymer PVP (Table S1 and Figure S1 in Supplementary Materials).

As evident from the electron density profiles shown in Figure 3, the addition of sterols increases the membrane thickness. Furthermore, the electron density of the sterol-containing samples is higher than that of pure POPE in the region 0.5–1.2 nm from the bilayer center (Figure 3). The differences in electron density shape suggest that the sterols are located within the hydrocarbon chain region of the lipid bilayer. In the two-component bilayer systems of PC/Chol, it is well known that Chol is distributed at this position [76].

According to reports by MacIntosh and Simon [77,78], the position of maximum electron density corresponds to the phosphate groups of the polar headgroups, and the point approximately 0.4 nm further outward corresponds to the outer edge of the lipid bilayer.

Using the membrane thickness determined in this manner and the apparent molecular volume ($V_{ap}$) obtained by the neutral flotation method described above, the average molecular area (*A*_0_) per lipid within the bilayer was calculated. The results are summarized in Table 1. As shown clearly in Table 1, Chol occupies a smaller surface area than Lan, indicating tighter molecular packing. This observation is consistent with trends observed in other phospholipid systems, demonstrating that Chol promotes tighter lipid packing [5,6]. The difference is likely due to the additional methyl group in Lan, which is less compatible with the geometry of phospholipid hydrocarbon chains.

### 3.3. Interbilayer Interaction

By subtracting the previously calculated membrane thickness from the lamellar repeat distance, the interbilayer distance—that is, the thickness of the water layer ($d_{w}$)—was determined. In calculating the electron density distribution, it was necessary to assign the phases (signs of the lamellar reflections). To achieve this, an experiment was performed using the neutral polymer PVP to apply osmotic pressure and thereby alter the thickness of the water layer. This experiment also provided information on how the interbilayer distance decreases under osmotic pressure, offering insight into interbilayer interactions [55].

**Figure 4:**
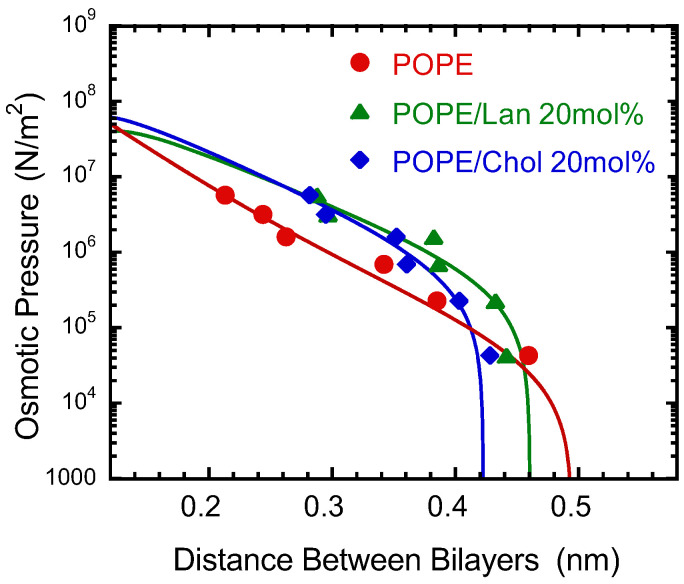
Applied osmotic pressure (logarithmic scale) plotted versus interbilayer distance (thickness of water layer) for pure POPE (red circle), POPE/Lan (green triangle), and POPE/Chol (blue diamond shape). For lines, see the body text.

Figure 4 shows the relationship between the interbilayer distance, i.e., the water layer thickness ($d_{w}$), and the applied osmotic pressure. The distance between lipid membranes in multilamellar vesicles is governed by the balance among three main forces: hydration repulsion [54,55,56], fluctuation-induced (entropic) repulsion [57,58,59] and van der Waals attraction [79].

Taking these three forces into account, the interbilayer pressure (*P*) is described by the following theoretical equation:

(3) $$P=P_{h}exp\left(-\frac{d_{w}}{\lambda_{h}}\right)+\frac{k_{B}T}{32\lambda_{w}}\sqrt{\frac{P_{h}}{K_{C}}}exp\left(-\frac{d_{w}}{2\lambda_{h}}\right)-\frac{H}{6\pi {d_{w}}^{3}},$$

where $P_{h}$ is a scaling constant, $\lambda_{h}$ is the decay length of the hydration force, *T* is the temperature, $K_{C}$ is the bilayer bending rigidity, and H is the Hamaker constant. This type of equation has also been used in previous studies [76,80,81,82], but for the final van der Waals attraction term, we used a simple inverse-cube-of-distance form based on reference [78].

We attempted to fit the experimental data using this equation. The fitting analysis was performed using the Fit command in Gnuplot (Version 5.4) (http://www.gnuplot.info/, 30 May 2025). However, when both the Hamaker constant and the membrane elasticity were allowed to vary simultaneously, the fitting procedure failed to converge. Therefore, we explored the most reasonable parameter values by fixing one parameter at a time. Even so, the asymptotic standard error values, which represent the 68% confidence interval for the estimated values output by Gnuplot, were approximately 10 to 20 times the values of the Hamaker constant and the membrane elasticity. Consequently, the fitting curve presented here should not be regarded as the best-fit solution but rather as a qualitative guide for interpreting the results. The parameters obtained tentatively from the fitting are listed in Table S2 of the Supplementary Materials for reference.

The results shown in Figure 4 indicate that, upon addition of sterols, the interbilayer distance (water layer thickness, $d_{w}$) increases under the same osmotic pressure in the range of $d_{w}=$ 0.2–0.4 nm. This suggests that sterols enhance the repulsive interactions between lipid bilayers, implying an increase in the hydration repulsion. The interpretation of this result will be discussed in the Discussion section.

Conversely, at larger interbilayer separations ($d_{w}>0.4$ nm), the presence of sterols leads to a reduction in spacing. One possible explanation involves the fluctuation-induced repulsive force, which constitutes a key component of interbilayer interactions. The incorporation of sterols stiffens the membranes, suppressing thermal fluctuations and thereby decreasing the interbilayer distance. This trend is consistent with results from electron density distributions and molecular volumes, indicating that Chol promotes tighter molecular packing and reduces membrane fluctuations. Similar behavior has been reported previously in phosphatidylcholine (PC) systems [81].

### 3.4. Structural Analysis of the H_II_ Phase

Structural analysis of the inverted hexagonal (H_II_) phase formed in mixtures of PE and sterols was performed. In this phase, lipid molecules arrange cylindrically around water channels, creating a hexagonal lattice. X-ray diffraction was used to analyze the H_II_ phase structure, and information about the lattice constants and lipid molecular arrangement was extracted from the diffraction patterns.

**Figure 5:**
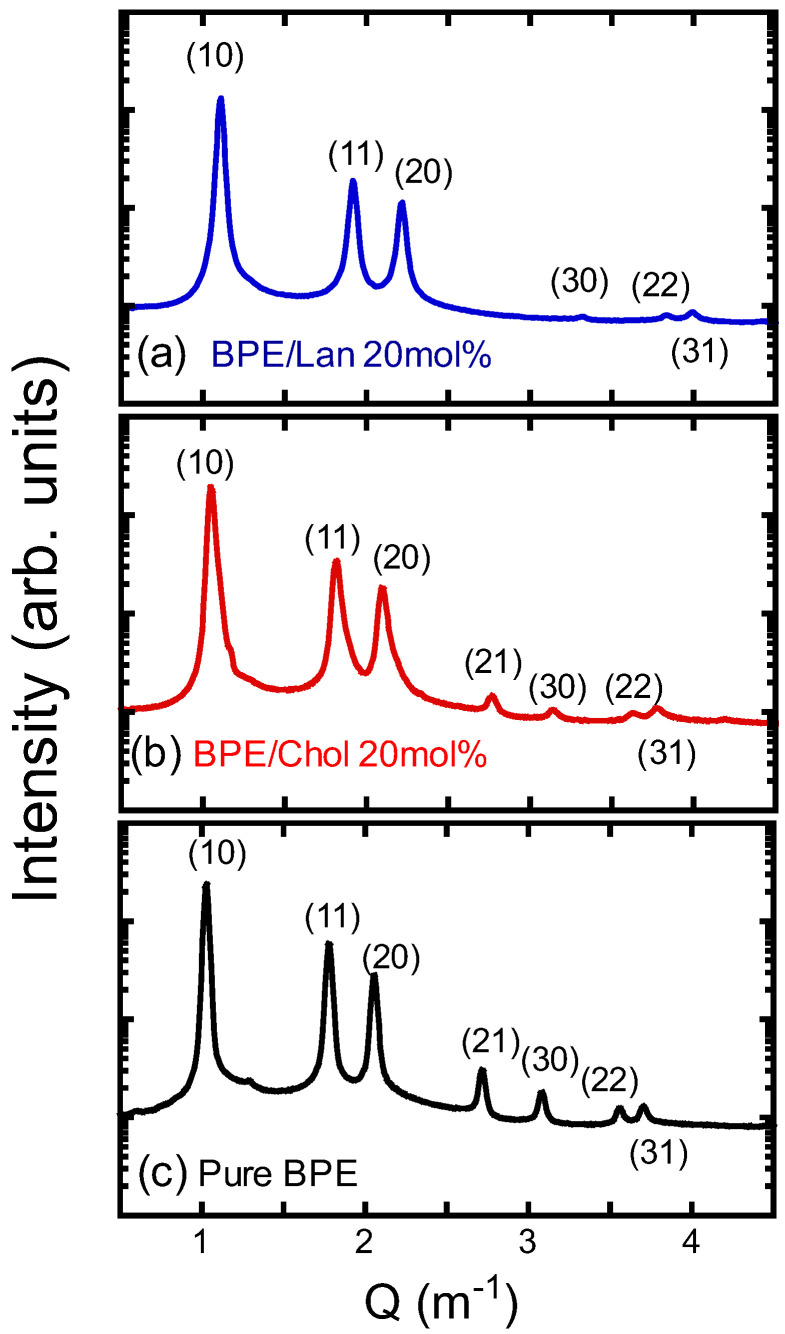
X-ray diffraction patterns of (**a**) BPE/Lan (20 mol%, (**b**) BPE/Chol (20 mol%), and (**c**) pure BPE. The patterns were recorded at 80 °C. The specified Miller index is shown above or below each diffraction peak.

Figure 5 shows representative small-angle X-ray diffraction patterns for the PE/sterol system—specifically, a sample containing 20 mol% sterol and pure bacterial PE (BPE). Each diffraction peak in the figure has been indexed, providing essential information for structural analysis. The positions of the observed diffraction peaks in *Q*-space followed the relationship $Q=\sqrt{h^{2}+k^{2}+hk}$, with the ratio being $1:\sqrt{3}:2:\sqrt{7}:3:2\sqrt{3}:\sqrt{13}$.

Instead of the POPE studied above, this experiment employed PE derived from the bacterium, *Escherichia coli*. Here, we denote it as BPE. When comparing POPE and BPE, they exhibit almost identical phase behavior. At 38 °C, both form a fluid lamellar phase, and at 80 °C, both form an H_II_ phase. This similarity in phase behavior is thought to arise from the composition of the main fatty acids in the hydrocarbon chains of BPE—palmitic acid (16:0) and oleic acid (18:1)—as described in the Materials and Methods section, which correspond to the two hydrocarbon chains of POPE. When using lipids with uniform hydrocarbon chain compositions, such as POPE, phase separation often occurred, preventing the formation of a homogeneous H_II_ structure. This was evidenced by multiple reflection peaks, indicating the coexistence of mixed periodic structures. Therefore, to achieve a well-defined H_II_ phase, BPE, which contains a heterogeneous distribution of hydrocarbon chains, was used. As a result, a clear diffraction pattern corresponding to a uniform H_II_ phase was obtained, as shown in Figure 5. This outcome is likely because the diversity of hydrocarbon chains promotes more entropic mixing of lipid molecules, leading to the formation of a stable H_II_ structure (hexagonal lattice) throughout the sample.

**Figure 6:**
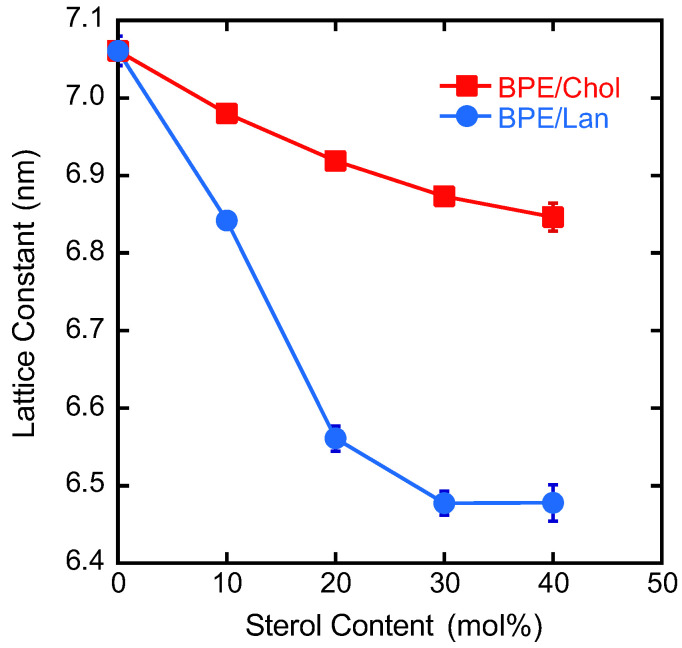
Plot of the lattice constants of the H_II_ phase of BPE/Chol (red square) and BPE/Lan (blue circle) as a function of sterol content.

The addition of sterols was found to affect the lattice constant. Specifically, an increase in the lattice constant was observed with increasing sterol concentration. The lattice constants calculated from the diffraction data are plotted as a function of sterol concentration in Figure 6. Similar to previous reports on the dielaidoyl-PE/Chol system [83], the lattice constants decreased with increasing Chol concentration. The addition of Lan also led to a decrease, which was more pronounced than the reduction observed with Chol.

**Figure 7:**
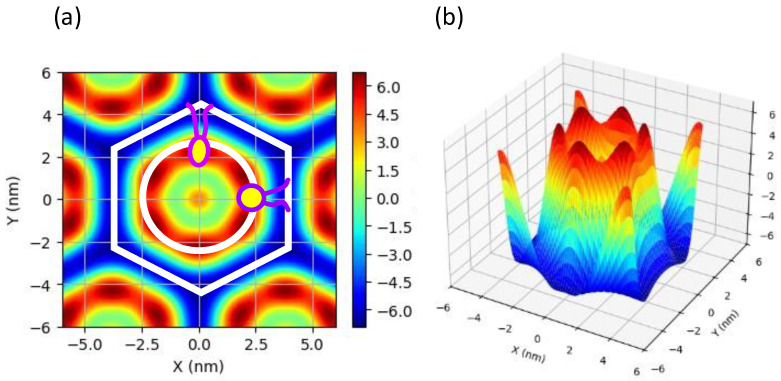
Relative electron density profile of pure BPE at 80 °C. (**a**) Two-dimensional map plot and (**b**) Three-dimensional plot. In (**a**), the white line represents the unit cell of a two-dimensional hexagonal lattice. The polar head position is also indicated schematically as a circle. The shape of the phospholipid molecule within the unit cell lattice is also depicted schematically. Note: This is a schematic representation only and does not accurately account for the size of the polar head group or the length of the hydrocarbon chain.

To obtain more detailed structural information, two-dimensional electron density maps were reconstructed from the diffraction intensity data. This analysis revealed the spatial arrangement of lipid molecules, the structure of the water channels, and sterol-induced modifications to the membrane architecture. Figure 7 shows the resulting electron density distribution for a pure BPE sample without sterols. Other results are displayed in Figure S3 of the Supplementary Materials.

The obtained electron density profile closely matches the two-dimensional distribution reported in previous studies on the H_II_ phase structure [69,70,71,72,73,74,75]. The regions of highest electron density correspond to the phosphate groups. Examination of the two-dimensional map confirms the presence of high-density regions along the circular boundaries, indicating that the polar headgroups and water interface form a relatively smooth curved surface.

**Figure 8:**
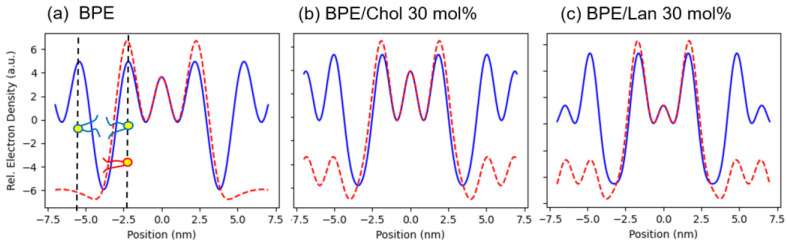
One-dimensional electron density profiles extracted in the horizontal (*X*) (blue lines) and vertical (*Y*) (red dashed lines) directions from a two-dimensional relative electron density distribution of the type shown in Figure 8. (**a**) pure BPE, (**b**) BPE/Chol (30 mol%), and (**c**) BPE/Lan (30 mol%).

In Figure 7, the schematic diagram shows that lipid molecules are arranged in a hexagonal lattice and that the thickness of the bilayer varies locally. Specifically, elongated lipid molecules are present in some regions, while compression is evident in others. To quantify this variation, electron density profiles were extracted along the X and Y axes (see Figure 8). Figure 8 shows the results for pure BPE and systems with a sterol concentration of 30 mol% as typical examples.

**Figure 9:**
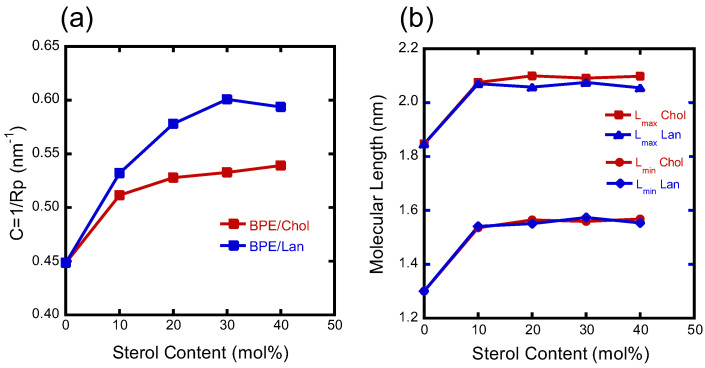
(**a**) Plot of sterol content versus the calculated curvature values of the H_II_ structure’s water column radii. BPE/Chol (red square) and BPL/Lan (blue square). (**b**) Plot of sterol content versus the maximum and minimum molecular lengths in the H_II_ structure. The red and blue data are from the BPE/Chol and BPE/Lan systems, respectively.

Figure 8 shows the results for pure BPE and systems with a sterol concentration of 30 mol% as typical examples. The results in Figure 8 demonstrate that the positions of maximum electron density coincide along both the *X*- and *Y*-directions, indicating that the water columns are nearly cylindrical. Based on this observation, the water column was modeled as a cylinder, and its radius and curvature were determined. The curvature values are plotted in Figure 9a.

The molecular length was also estimated from the electron density distribution. It was calculated from the distance between the edge of the hexagonal lattice (indicated in white in Figure 7) and the center of the cylindrical region, yielding the maximum and minimum molecular lengths. The results are presented in Figure 9b.

These results indicate that, when comparing Chol and Lan, Lan induces greater curvature, suggesting that it promotes more flexible molecular packing. However, no significant difference in molecular length was observed between the two sterols.

## 4. Discussion

First, we discuss the molecular volume (Figure 1). At the temperatures studied here, pure Chol and Lan exist in a solid state. Note that the linearity in Figure 1 does not imply ideal mixing of sterols in the solid state and the fluid POPE bilayer state. The molecular volume of Chol, calculated from its crystal structure, is larger than the molecular volume it occupies in the POPE bilayer (its apparent molecular volume), as estimated by extrapolation from Figure 1. This discrepancy arises because the molecular shape of cholesterol is well-suited to fitting into the flexible hydrocarbon chain environment in the melt, as discussed in our previous paper [45]. The linear trend in Figure 1 indicates that there is no concentration-dependent change in the interaction between sterols and POPE within the investigated sterol concentration range and that no phase boundary exists in the phase diagram within this range. In contrast, in mixtures of dimyristoylphosphatidylcholine and dipalmitoylphosphatidylcholine with Chol, both of which contain saturated fatty acid chains, bending was observed at the phase boundary.

The calculated structural parameters of pure POPE and the POPE/sterol systems containing 20 mol% sterol, based on the apparent molecular volumes determined by the neutral flotation method and the X-ray structural analysis, are summarized in Table 1. As shown in this table, the apparent molecular volume of the Lan system is larger than that of the Chol system. This is reasonable, as Lan possesses three additional methyl groups compared to Chol. However, the membrane thickness of the Chol system is greater than that of the Lan system, while the surface area per molecule is smaller. This reflects the denser lateral packing of the membrane, which leads to elongation along the normal direction to the membrane surface. In other words, the Chol-containing membrane is more tightly packed and thicker. From the viewpoint of enhancing membrane barrier properties, Chol can be regarded as a more evolutionarily optimized molecule than Lan. This observation is consistent with Bloch’s hypothesis [28,29,30] on the molecular evolution of sterols and agrees with many previous experimental findings in phosphatidylcholine (PC) systems [31,32,33,34,35,36,37,38,39,40,41,42,43,44,45].

As shown in Figure 1, within the accuracy of our measurements, the apparent molecular volume can be fitted to a linear function with a negative slope as a function of sterol concentration. If a strong attractive interaction existed between Chol and phospholipids, the plot in Figure 1 would be expected to exhibit a concave-downward shape, as reported in single-molecule experiments. Bloch [29] proposed that van der Waals interactions might be stronger for Chol than for Lan; however, no evidence supporting this was obtained in the present study. The experimental results can be interpreted primarily in terms of molecular shape: Chol, with its smoother, slimmer structure, fits more snugly with the phospholipid hydrocarbon chains than Lan.

As seen in the electron density profiles shown in Figure 3, both Chol and Lan are located within the hydrocarbon region, relatively far from the polar headgroups. Thus, the influence of the headgroups is expected to be weak. This is supported by the experimental results that the occupied molecular volumes of Chol and Lan in POPE membranes, extrapolated from the data, are nearly identical to those in POPC membranes (Figure 1).

In PE bilayer systems, McIntosh and Simon [78] experimentally demonstrated that when the interbilayer distance is relatively short, an additional attractive interaction exists in addition to the van der Waals force (a result also suggested by molecular dynamics simulations [84]). They proposed that this attraction may arise from direct electrostatic interactions between the positively charged amine and the negatively charged phosphate groups of PE, or from indirect hydrogen bonding mediated by water molecules, depending on the charge state. The addition of sterols to POPE decreases the number of polar headgroups per unit membrane area; therefore, sterols should weaken the attractive forces associated with headgroup interactions. Indeed, as shown in Figure 4, when the applied osmotic pressure is in the range of 10^5^–10^7^ N/m^2^, the intermembrane distance increases upon sterol addition under the same osmotic pressure. This observation supports the above interpretation.

For PC systems, it has been reported that the addition of Chol weakens the repulsive force at short distances ($d_{w}$ = 0.2–0.4 nm) [85]. Unlike PE, PC has relatively large polar head groups, and at such short distances, the steric repulsion that occurs when the polar head groups come into physical contact with each other is strong. This is interpreted as being due to the fact that the addition of Chol reduces the number of PC polar head groups per membrane surface area, thereby weakening this steric repulsion [85]. The effect of sterol addition on interlayer interactions appears to differ between PE and PC.

Since sterols increase the repulsive force between bilayers at short distances on PE systems, it is possible to hypothesize that Chol plays a role in preventing uncontrolled membrane fusion in PE-rich regions of cell and organelle membranes.

Conversely, at osmotic pressures below 10^5^ N/m^2^, where the intermembrane distance is relatively large, the addition of sterols reduces the intermembrane spacing. The order of the reduction is POPE/Chol < POPE/Lan < pure POPE. As pointed out by Petrache et al. [81], the incorporation of sterols thickens and stiffens membranes through denser molecular packing, which suppresses thermally induced membrane undulations and thereby reduces the so-called undulation (entropic) repulsion. Consistent with the partial volume analysis above, our results indicate that Chol suppresses membrane fluctuations more effectively than Lan. The reduction in membrane fluctuations suppresses the repulsive forces between bilayer membranes that arise from them.

When membranes are relatively far apart, the presence of sterols tends to draw them closer together; however, at short separations—just before membrane contact—the sterols act in a repulsive manner, potentially hindering direct fusion. Therefore, it is difficult to determine whether the presence of sterols promotes or inhibits membrane fusion solely from experiments on intermembrane interactions in such simplified model systems.

Finally, we discuss the role of sterols in membrane fusion from the perspective of H_II_ phase formation. Compared to Chol, Lan decreases the radius of the water channel in the H_II_ phase of BPE, thereby increasing the curvature of the water–lipid interface (Figure 9a). From the standpoint of the molecular packing parameter, which depends on the molecular length, hydrocarbon chain volume, and headgroup size, Chol has a larger molecular volume than Lan. Because both sterols are embedded within the hydrocarbon region, it is reasonable to expect that the Lan system possesses a larger packing parameter and forms an H_II_ phase with higher curvature. From this viewpoint, Lan appears to be more favorable for membrane fusion. However, in terms of intermembrane interactions, Lan has a weaker effect in suppressing membrane fluctuations, suggesting that Chol is more advantageous for fusion. Further investigations are required, as membrane fusion involves multiple contributing factors.

## 5. Conclusions and Perspectives

In this work, we examined the effects of cholesterol (Chol) and its biosynthetic precursor lanosterol (Lan) on hydrated phosphatidylethanolamine (PE) membranes to clarify how sterol structure influences interbilayer interactions and curvature formation relevant to membrane fusion. Volumetric measurements and X-ray diffraction analyses revealed that Lan possesses a larger molecular volume than Chol. Extrapolation of the occupied molecular volumes of Chol and Lan in phospholipid bilayers showed no difference between POPC and POPE, indicating deep insertion of sterols within the hydrophobic region, independent of the phospholipid headgroup type. Both sterols increased membrane thickness and strengthened short-range hydration repulsion between bilayers. However, Chol suppressed fluctuation-induced repulsion more efficiently, consistent with its stronger membrane-stiffening ability. Structural analysis of the inverted hexagonal (H_II_) phase further showed that Lan induced greater curvature of the water channels compared with Chol, suggesting that Lan promotes more flexible molecular packing within the membrane. These observations imply that Chol primarily contributes to membrane stabilization through enhanced rigidity and reduced fluctuations, whereas Lan may facilitate curvature required for fusion intermediates. Taken together, the results highlight that small structural variations among sterols can lead to distinct mechanical and morphological effects on lipid membranes. Such differences may underlie the evolutionary optimization of Chol as a molecule that enables fine regulation of membrane dynamics and fusion processes in biological systems.

The resolution of the experimental data here did not permit a precise determination of sterol location in phospholipid assemblies. Future studies using small-angle neutron scattering with deuterated sterols and contrast-varying methods may effectively address this issue. In theory, parameters related to the lipid membrane’s elastic properties can be calculated from analysis of X-ray diffraction data using Equation (3). However, the quality of the present data did not permit the determination of reliable values. Instead of using indirect methods, one option would be to evaluate viscoelasticity using a quartz crystal microbalance with dissipation monitoring (QCM-D) [86].

## Figures and Tables

**Table 1:** Structural parameters of pure POPE and POPE containing 20 mol% sterols at 38 °C.

	$D_{B}$ (nm)	$V_{ap}$ (nm^3^)	*A*_o_ (nm^2^)
POPE	4.78	1.177	0.492
POPE/Chol	4.92	1.067	0.434
POPE/Lan	4.88	1.080	0.443

## Data Availability

Data will be made available on request.

## Supplementary Materials

The following supporting information can be downloaded at: https://www.mdpi.com/article/10.3390/membranes15120352/s1, Figure S1: Two typical examples of row diffraction images; Figure S2: Normalized structure factors calculated from the observed lamellar diffraction intensity data are plotted against the scattering vector magnitude (*Q*); Figure S3: Two-dimensional relative electron density profile plots of the H_II_ phase structures; Table S1: Lamellar repeat distance (*D* (nm)) and relative diffraction intensity of each Bragg peak (*I*_n_); Table S2: Fitting parameters of the pressure-distance curve (Figure 4) for pure POPE and POPE containing 20 mol% sterols at 38 °C. This supplementary material cites references [66,67,87].

## Abbreviations

The following abbreviations are used in this manuscript:

Chol	Cholesterol
Lan	Lanosterol
SARS-CoV-2	Severe acute respiratory syndrome coronavirus 2
POPE	1-Palmitoyl-2-oleoyl-sn-glycero-3-phosphoethanolamine
POPC	1-Palmitoyl-2-oleoyl-sn-glycero-3-phosphocholine
BPE	L-α-Phosphatidylethanolamine from the bacterium Escherichia coli
PC	Phosphatidylcholine
PE	Phosphatidylethanolamine
HII	Inverted hexagonal
PVP	Polyvinylpyrrolidone
