# Supplementary material for: A Comparative Study of the Effects of Cholesterol and Lanosterol on Hydrated Phosphatidylethanolamine Assemblies: Focusing on Physical Parameters Related to Membrane Fusion
Source: Membranes (Basel). 2025 Nov 24;15(12):352. doi: 10.3390/membranes15120352 (PMC12734454; doi:10.3390/membranes15120352)
Supplement: Supplementary file 1 [file membranes-15-00352-s001.zip › membranes-3987278-supplementary.pdf]

Supplementary Materials

A Comparative Study of the Effects of Cholesterol and Lanosterol on Hydrated Phosphatidylethanolamine Assemblies: Focusing on Physical Parameters Related to Membrane Fusion

Ayumi Okayama [1,] Michael Postrado [1] and Hiroshi Takahashi [1,*]

[1] Division of Pure and Applied Science, Faculty of Science and Technology, Gunma University, 4-2 Aramaki, Maebashi, Gunma 371-8510, Japan.

* Correspondence: hirotakahashi@gunma-u.ac.jp; Tel.: +81-27-220-7552

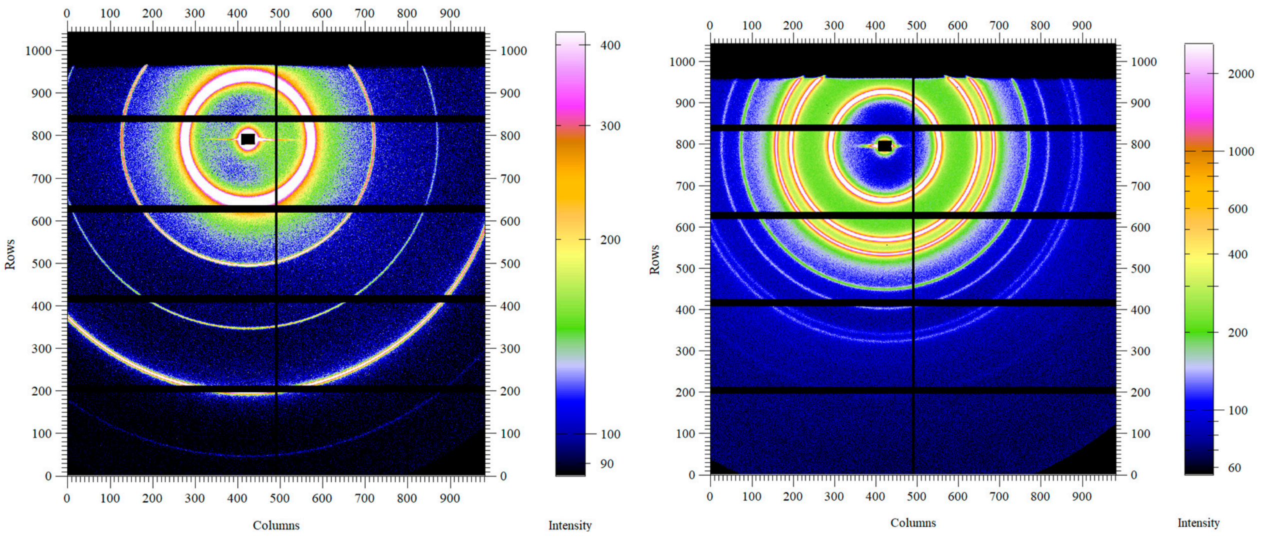

Figure S1. Two typical examples of raw two-dimensional diffraction images recorded with the PILATUS detector without subtracting background scattering: (left) Lamellar phase, POPE/Chol (20 mol%) in 20 wt% PVP solution recorded at 38 °C; (right) H_{II} phase, BPE/Chol (10 mol%) in pure water recorded at 80 °C.

Table S1. Lamellar repeat distance (D (nm)) and relative diffraction intensity of each Bragg peak (I_n) in arbitrary units*.

(a) purePOPE

	PVP 0 wt%	PVP 10 wt%	PVP 20 wt%	PVP 30 wt%	PVP 40 wt%	PVP 50 wt%	PVP 60 wt%
D (nm)	5.23	5.22	5.19	5.16	5.06	5.04	4.99
I_1 (a.u)	1864	31166	24181	30579	17734	13510	21957
I_2 (a.u)	7.09	86.20	55.08	33.88	1.81	1.09	26.90
I_3 (a.u)	8.97	156.75	111.93	115.87	26.26	16.95	4.090
I_4 (a.u)	9.78	237.16	173.92	217.38	112.41	80.06	107.52
I_5 (a.u)	0.09	1.50	2.45	3.40	2.61	2.34	2.91

(b) POPE/Chol 20 mol%

	PVP 0 wt%	PVP 10 wt%	PVP 20 wt%	PVP 30 wt%	PVP 40 wt%	PVP 50 wt%	PVP 60 wt%
D (nm)	5.40	5.39	5.36	5.30	5.28	5.18	5.14
I_1 (a.u)	1863	811	3179	2870	1936	3563	985
I_2 (a.u)	7.09	4.33	11.83	5.47	3.24	0.24	0.17
I_3 (a.u)	8.97	1.36	2.28	0.55	0.31	0.70	0.49
I_4 (a.u)	9.78	3.17	19.56	17.91	11.67	18.47	4.14
I_5 (a.u)	0.18	0.43	1.05	0.90	0.48	0.80	0.00

POPE/Lan20 mol%

	PVP 0 wt%	PVP 10 wt%	PVP 20 wt%	PVP 30 wt%	PVP 40 wt%	PVP 50 wt%	PVP 60 wt%
D (nm)	5.34	5.33	5.31	5.25	5.17	5.12	5.08
I_1 (a.u)	2006	1592	1746	1981	1314	2394	1718
I_2 (a.u)	9.57	6.86	6.35	2.83	0.75	0.40	0.72
I_3 (a.u)	4.85	2.39	2.57	0.80	1.15	1.13	0.54
I_4 (a.u)	10.53	10.18	9.00	10.57	8.28	10.88	7.44
I_5 (a.u)	0.24	0.23	0.20	0.00	0.00	0.31	0.21

*The intensities in this table cannot be used to compare diffraction intensities between different samples. To calculate structure factors, normalization was performed using the relation

$$\left(\sum I_n\right)/D = \text{constant}$$

, according to the method proposed previously[87].

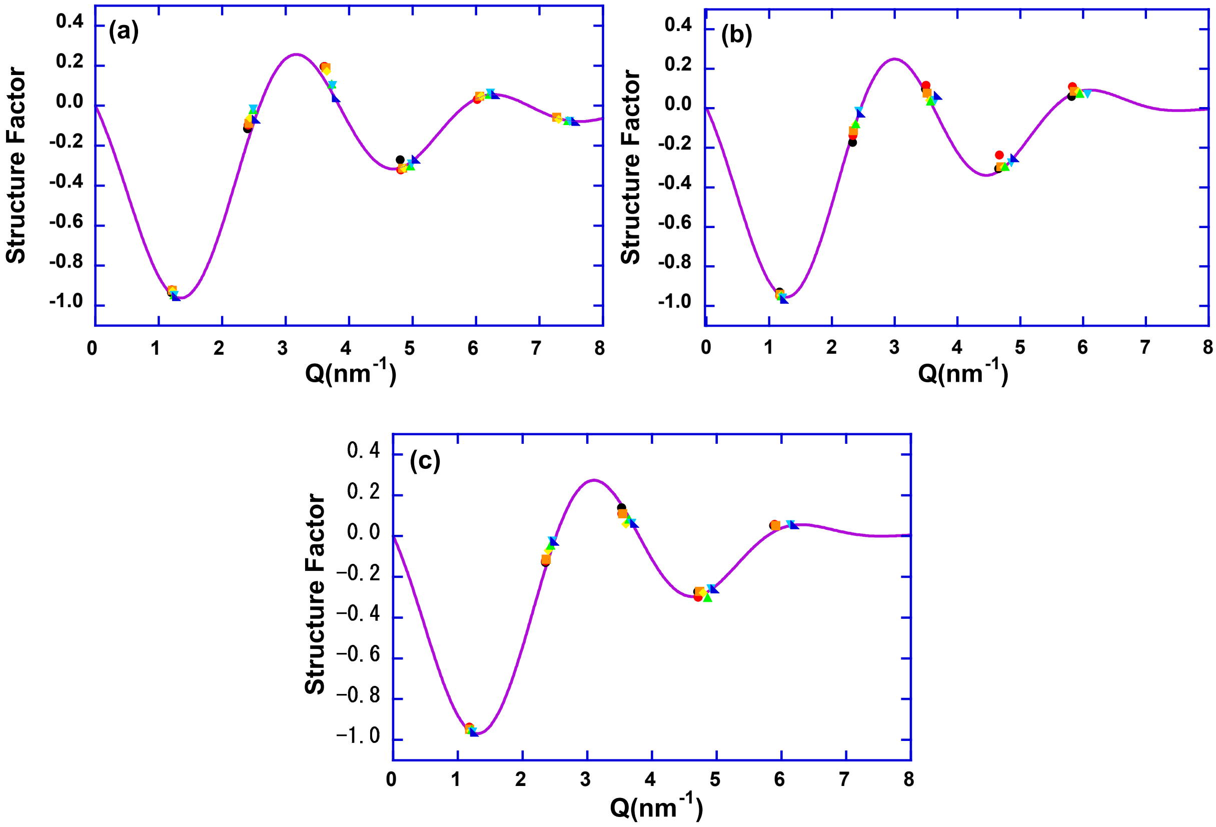

Figure S2. Normalized structure factors calculated from the observed lamellar diffraction intensity data are plotted against the scattering vector magnitude (Q). (a) pure POPE (b) POPE/Chol, and POPE/Lan. The solid curves are the continuous Fourier transform calculated using Shannon's sampling theorem with the data obtained in the presence of PVP 30wt% ((a) and (c)) or PVP 60wt% (b) [66,67]. For each data point, ●, ●, ■, ◆, ▲, ▼, ◣, represent the structural factors PVP0wt%, PVP10wt%, PVP20wt%, PVP30wt%, PVP40wt%, PVP50wt%, PVP60wt%, respectively.

Table S2. Fitting parameters of the pressure-distance curve (Figure 4) for pure POPE and POPE containing 20 mol% sterols at 38 °C.

	P_h (10^9 N/m^2)	λ_h (10^{-2} nm)	K_C (10^{-19} J)	H(10^{-21} J)
POPE	1.13	3.7	0.17	0.15
POPE/Chol	1.21	5.2	1.6	3.0
POPE/Lan	0.90	5.4	0.9	3.0

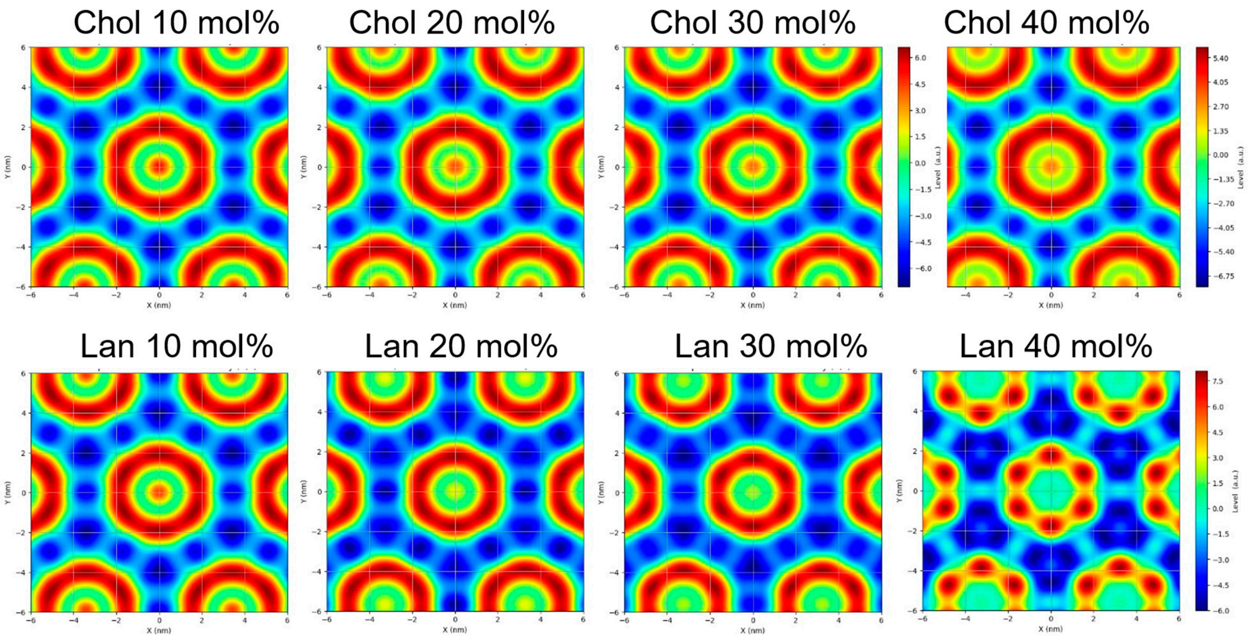

Figure S3. Two-dimensional relative electron density maps of the H_{II} phase structures. The upper and lower sets of figures show the electron density profiles of POPE/Cho and POPE/Lan, respectively. Sterol concentrations are indicated above each map.

References

66. Sayre, D. Some implications of a theorem due to Shannon. *Acta. Cryst*. **1952**, *5*, 843. https://doi.org/10.1107/S0365110X52002276.
67. Franks, N.P. Structural analysis of hydrated egg lecithin and cholesterol bilayers. I. X-ray diffraction. *J. Mol. Biol.* **1976**, *100*, 345–358. https://doi.org/10.1016/s0022-2836(76)80067-8.
87. Worthington, C.R., Blaurock, A.E.; A structural analysis of nerve myelin. *Biophys. J*. **1969**, 9, 970-90. https://doi.org/10.1016/S0006-3495(69)86431-3.
